# Further phenotypic delineation of the auriculocondylar syndrome type 2 with literature review

**DOI:** 10.1007/s13353-020-00591-3

**Published:** 2020-10-31

**Authors:** Ewelina Bukowska-Olech, Anna Sowińska-Seidler, Filip Łojek, Delfina Popiel, Joanna Walczak-Sztulpa, Aleksander Jamsheer

**Affiliations:** 1 Department of Medical Genetics, Poznan University of Medical Sciences, Rokietnicka 8 Street, 60-806 Poznan, Poland; 2 Centers for Medical Genetics GENESIS, Dąbrowskiego 77A Street, 60-529 Poznan, Poland

**Keywords:** Next-generation sequencing, PLCB4, Question mark ear, Auriculocondylar syndrome

## Abstract

Auriculocondylar syndrome (ACS) is an ultra-rare disorder that arises from developmental defects of the first and second pharyngeal arches. Three subtypes of ACS have been described so far, i.e., ACS1 (MIM: 602483), ACS2 (MIM: 600810), and ACS3 (MIM: 131240). The majority of patients, however, are affected by ACS2, which results from the mutations in the *PLCB4* gene. Herein, we have described an 8-year-old male patient presenting with ACS2 and summarized the molecular and phenotypic spectrum of the syndrome. We have also compared the clinical features of our case to three other previously described cases (one sporadic and two familial) harboring the same heterozygous missense variant c.1862G>A, p.Arg621His in the *PLCB4* gene. The mutation was detected using whole-exome sequencing (WES). Due to low coverage of WES and suspicion of somatic mosaicism, the variant was additionally reassessed by deep targeted next-generation sequencing panel of genes related to the craniofacial disorders, and next confirmed by Sanger sequencing. ACS2 presents high intra- and interfamilial phenotypic heterogeneity that impedes reaching an exact clinical and molecular diagnosis. Thus, describing additional cases, carrying even the known mutation, but resulting in variable phenotypes, is essential for better understanding of such orphan Mendelian diseases.

**Supplementary Information:**

The online version contains supplementary material available at 10.1007/s13353-020-00591-3.

## Introduction

Auriculocondylar syndrome (ACS), also known as question mark ear syndrome or dysgnathia complex, is an ultra-rare disorder that belongs to a group of malformations arising from the developmental defects of the first and second pharyngeal arches (Passos-Bueno et al. 2009). Three subtypes of ACS have been described so far, i.e., ACS1 (MIM: 602483), ACS2 (MIM: 600810), and ACS3 (MIM: 131240), resulting from deleterious variants within *GNAI3* encoding G protein subunit alpha i3, *PLCB4* encoding phospholipase C beta 4, and *EDN1* encoding endothelin 1, respectively (Rieder et al. 2012; Gordon et al. 2013a, b). Noteworthy, all of those genes constitute the EDN1-EDNRA pathway, which contributes to craniofacial development through induction, migration, and maintenance of neural crest cells (Bonano et al. 2008).

The clinical diagnosis of ACS may be challenging due to its high intra- and interfamilial phenotypic variability and infrequent occurrence, which is estimated to be < 1 per 1,000,000 (Nabil et al. 2020; Storm et al. 2005). However, the core features include the variable degree of micrognathia, often associated by temporomandibular joint ankylosis, cleft palate, and distinctive ear malformations in the form of cleft or notch within the helix, giving the appearance of a question mark. Other frequently noted features comprise facial asymmetry, hearing impairment, prominent cheeks, preauricular tags, and microstomia (Gordon et al. 2015; Romanelli Tavares et al. 2017).

Herein, we report the next case of ACS2 and summarize the molecular and phenotypic spectrum of the syndrome. We have also compared the clinical features of an 8-year-old patient to three other previously described cases (one sporadic and two familial), harboring the same heterozygous missense variant c.1862G>A, p.Arg621His in the *PLCB4* gene. The mutation was detected using whole-exome sequencing (WES). Due to low coverage and suspicion of somatic mosaicism in WES analysis, the variant was additionally assessed by deep targeted next-generation sequencing (NGS), and confirmed by Sanger sequencing.

## Methods

### GTG banding and microarray analysis

We performed karyotyping on peripheral blood lymphocytes of the index patient using the Giemsa-banding technique at 550 band resolution per haploid genome. Moreover, array comparative genomic hybridization (aCGH) (SurePrint G3 Human CGH Microarray 8×60k; Agilent Technologies) was performed on the high-quality genomic DNA (gDNA) of the index. gDNA was extracted from the peripheral blood lymphocytes using the MagCore® HF16 automated nucleic acid extractor (RBC Bioscience Corp.).

### Whole-exome sequencing

Whole-exome sequencing (WES) was performed in the proband and his healthy parents using the Sure Select Enrichment Kit (Agilent Technologies) and Illumina Paired-End Sequencing Kit (Agilent Technologies) followed by sequencing on Illumina HiSeq 1500. The sequenced reads were mapped to the GRCh38 human reference genome. A variant discovery pipeline was built based on the GATK Best Practices. In order to extract potentially causative variants, we applied a Phenotypic Interpretation of eXomes (PhenIX) computational algorithm using the following Human Phenotype Ontology (HPO) terms: micrognathia (HP: 0000347), atresia of the external auditory canal (HP: 0000413), and microtia (HP: 0008551) (Jamsheer et al. 2016).

### Targeted next-generation sequencing

We used the custom On-Demand AmpliSeq (Thermo Fisher Scientific) gene panel with 2 primer pools and sequenced 37 genes related to the craniofacial disorders, including *PLCB4* (Supplementary File 1) (Bukowska-Olech et al. 2020)*.* The barcoded gDNA libraries were constructed according to the manufacturer’s sample preparation protocol (Ion AmpliSeq Library Kit 2.0; On-Demand Panels) and subsequently sequenced on the Ion Torrent S5 platform with the use of the Ion 540™ Kit. The sequenced reads were mapped to the GRCh38 human reference genome, and the variant of interest (chr20:9389727) was analyzed applying the Integrative Genomics Visualization (IGV) tool.

### Sanger sequencing

We performed the validation of the variant using PCR and conventional Sanger sequencing. Specific primers were designed with the use of the Primer3 tool v. 0.4.0 (http://bioinfo.ut.ee/primer3-0.4.0/). Primer sequences 5′–3′ were as follows: gctgtgacaatctgcccaaa (forward), ggcaccatcctgtcaaagtg (reverse). The PCR and PCR product purification were carried out following standard protocols. Sanger sequencing was performed on the automated sequencer Applied Biosystems Prism 3700 DNA Analyser using the dye-terminator chemistry kit v.3, ABI 3130XL. Next, the variant was visualized by the BioEdit tool and annotated against the reference *PLCB4* sequence NM_000933.3 following the Human Genome Variation Society (HGVS) nomenclature guidelines.

### In silico analysis

The 3D structure of the PLCB4 mutation site was predicted using the SWISS-MODEL homology modeling server (Waterhouse et al. 2018). As a template, the rat phosphoinositide-specific phospholipase C, isozyme delta1 (1djz.2.A) structure was used (Essen et al. 1997). The template had a 37.67% sequence homology with the human PLCB4, had an X-ray crystal structure of high resolution (3.0 Å), and contained 1D-myo-inositol-4,5-bisphosphate (IP2) ligand. The modeling was performed for the wild-type and mutated protein. Conservation analysis was done applying the Varsome tool (https://varsome.com/) and Alamut® Visual software.

### Clinical report

**Fig. 1:**
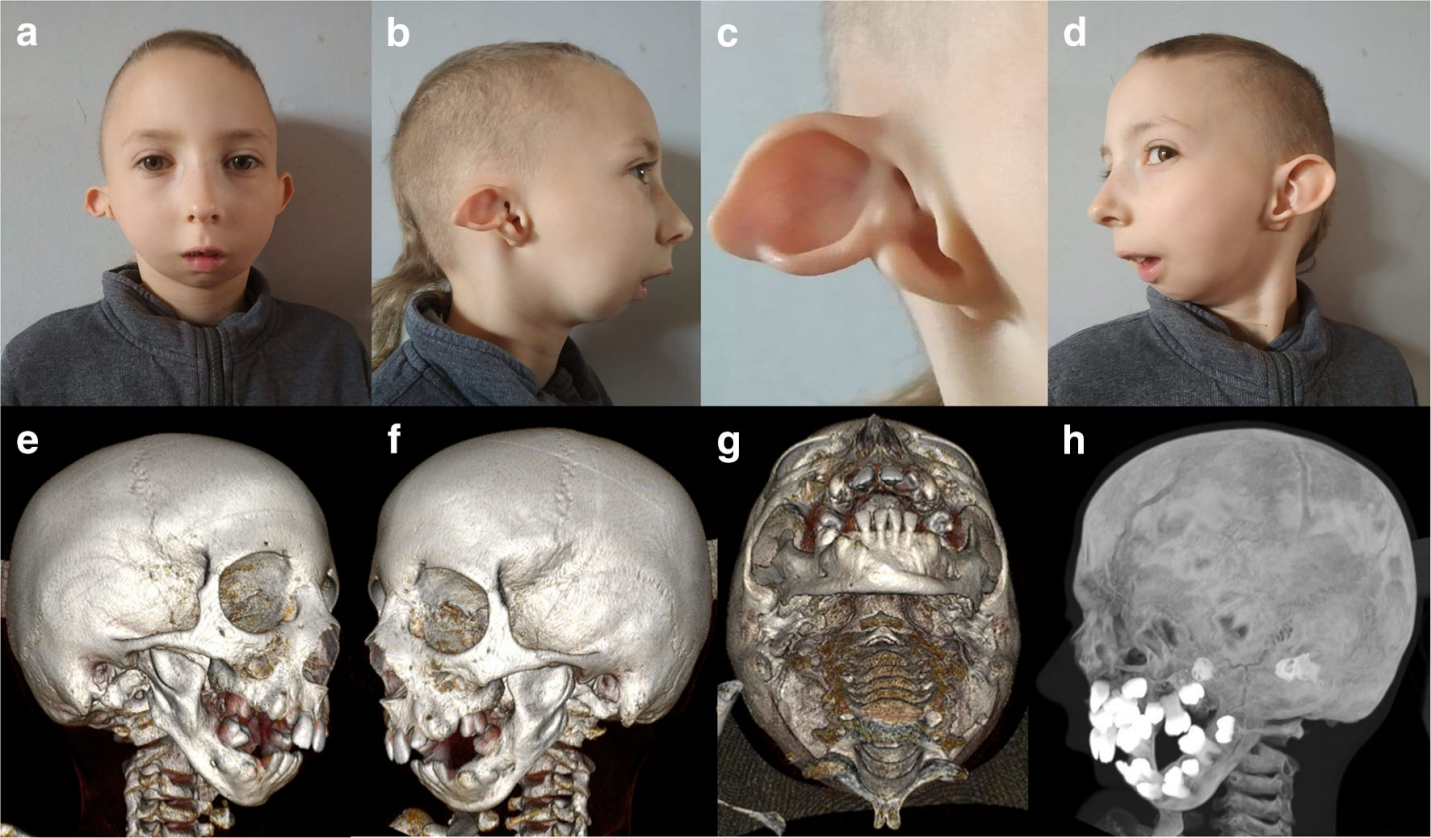
Phenotypic presentation of the index patient. **a** Anterior posterior view of the face showing full cheeks and round facial shape. **b**, **d** Lateral view of the face. **b**, **c** The notch within the right helix, giving the appearance of a question mark ear. **e**–**h** A 3D reconstruction of a computational tomography indicating micrognathia, with mandibular hypoplasia more pronounced on the left side. Furthermore, coronoid processes and mandibular condyles are significantly hypoplastic on both sides. Lateral view of the patient’s skull shows crowded teeth in the upper and lower jaws

The 8-year-old male patient was born to unrelated and healthy parents in the 40^th^ week of gestation by Cesarean section from the 5^th^ pregnancy complicated with threatened abortion. His body mass was 2860 g (10^th^ percentile), length 53 cm (75^th^–90^th^ percentile), and head circumference 31 cm (below 3^rd^ percentile), while the Apgar score was 10 at 1′. After birth, marked mandibular hypoplasia (micro- and retrognathia), facial asymmetry, atresia of the right external auditory canal, and asymmetric hypoplasia of both pinnae (more severe on the right side) were observed. Psychomotor development was within normal range, with independent sitting achieved at 6 months, and walking at 18 months of age. The patient spoke first words at 12 months of age, but further speech development was delayed due to conductive hypoacusis. During the last clinical evaluation at the age of 8 years, the index had normal intellectual development, but manifested aggressive behavior. His abdominal ultrasound scan was inconspicuous. The main clinical features described during the genetic evaluation are listed in Table 1 and Fig. 1.

**Table 1:** The comparative analysis of clinical features present in the index case vs. reported patients with auriculocondylar syndrome 2 caused by c.1862G>A, p.Arg621His *PLCB4* alteration

	Index case	M001	Case 4	Family 1 Case III: 2	Family 1 Case II: 4	Family 1 Case III: 1	Family 1 Case II: 1
Submitter		Rieder et al.	Gordon et al.	Nabil et al.	Nabil et al.	Nabil et al.	Nabil et al.
Occurrence	Sporadic	Familial	Sporadic	Familial	Familial	Familial	Familial
Gender	M	M	M	F	M	M	M
Micrognathia and/ or retrognathia	+	+	+	+	+	+	+
Ankylosis	−	−	?	+	?	?	+
Cleft palate	−	−	−	−	−	−	−
Ear malformation	+	+	+	+	+	+	+
Prominent cheeks	+	+	+	+	+	+	+
Auricular tags	−	+	+	−	−	−	−
Microstomia	+	+	+	+	−	−	+
Mastication difficulties	+	+	+	+	−	−	+
Speech articulation difficulties	+	?	+	+	−	−	+
Obstructive apnea	−	?	?	+	−	−	+
Snoring	+	?	?	+	−	−	+
Crowded teeth	+	?	−	+	−	+	−
Round face	+	+	+	+	+	−	+
Facial asymmetry	+	+	+	+	+	+	+
Mandibular condyle hypoplasia	+	?	+	+	?	?	+
Asymmetric mandible	+	+	+	+	+	+	+
Short mandibular rami	+	?	+	+	?	+	+
Additional feature	−	Absence of facial hair	Neonatal hypotonia, developmental delay	Unilateral thymic cyst	Absence of facial hair	Absence of facial hair	Right neck lesion

## Results

### GTG banding and microarray analysis

Conventional chromosome analysis (GTG banding) based on the patient’s peripheral blood lymphocytes showed normal male karyotype. Microarray analysis with the use of 60k 8-plex array (SurePrint G3 Human CGH, Agilent) detected no copy number variations larger than 100 kb in proband’s whole-blood DNA.

### Next-generation and Sanger sequencing methods

**Fig. 2:**
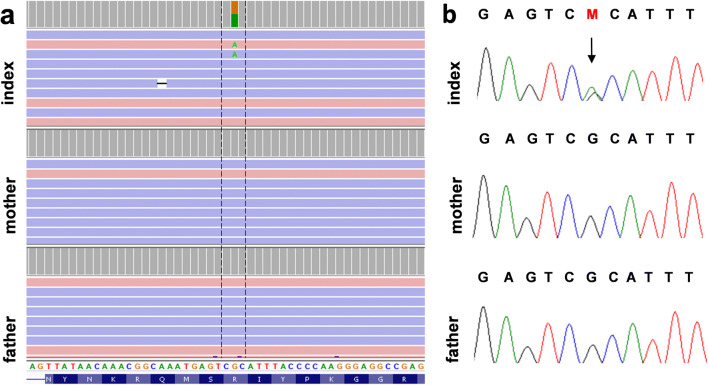
The molecular results in the index patient. The representation of the c.1862G>A *PLCB4* variant revealed in heterozygosity in the index (**a - upper image**) and excluded in both parents (**a** - mother & father in the middle and bottom image, respectively) employing targeted next-generation sequencing. Chromatograms show targeted validation studies of the index patient (**b - upper image**) and his parents (**b** - middle and bottom images) performed with the use of Sanger sequencing

WES revealed that the affected individual carried the heterozygous variant c.1862G>A, p.Arg621His in the 20^th^ exon of *PLCB4* (hg38; chr20:9409080, RefSeq NM**_**000933.3), whereas both of his healthy parents were negative for this variant. The missense mutation was reported in the Human Gene Mutation Database (HGMD; CM123396), Online Mendelian Inheritance in Man (OMIM; 600810.0003), ClinVar (accession: VCV000031639.1; variation ID: 31639), and dbSNP (rs387514481) databases. Due to low coverage and suspicion of somatic mosaicism in WES, the alteration was additionally assessed by deep targeted next-generation sequencing. In this step, we achieved 2177 reads for the analyzed variant. Our result demonstrated heterozygosity of the mutation since the reference adenine nucleotide was called in 1070 reads, whereas mutated guanine in 1096 reads. The alteration was subsequently confirmed and shown to be heterozygous by Sanger sequencing (Fig. 2).

### 3D modeling of the PLCB4 protein mutation site and conservation analysis

**Fig. 3:**
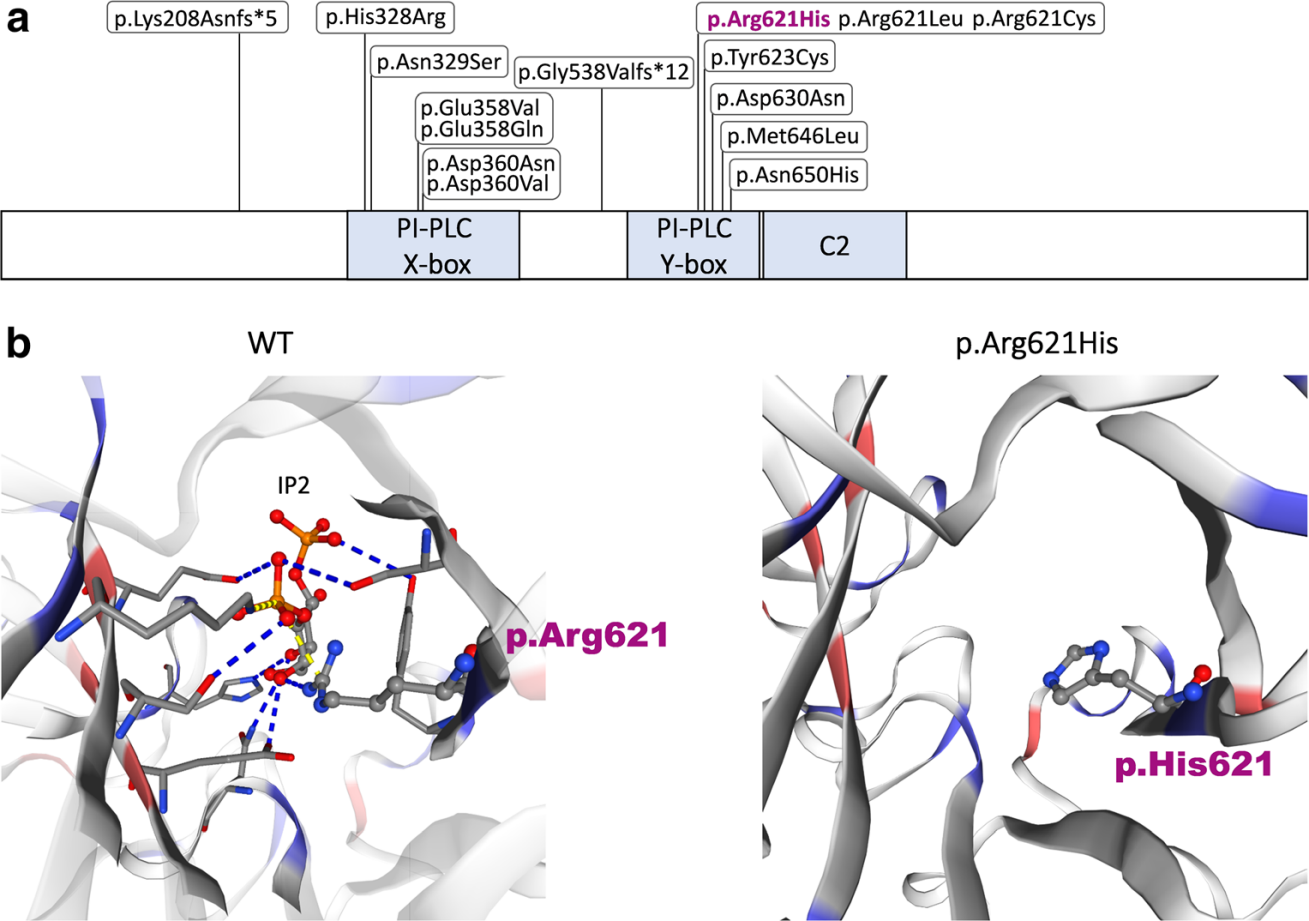
**a** The PLCB4 protein structure. Schematic representation of the PLCB4 protein with marked mutations reported in ACS2. PI-PLC X-box - phosphatidylinositol-specific phospholipase C X domain; PI-PLC Y-box - phosphatidylinositol-specific phospholipase C Y domain; C2 - C2 domain. **b** 3D structure prediction of the active site of wild-type (WT) and mutated (Arg621His) PLCB4. Hydrogen bonds and salt bridges between amino acid residues and 1D-myo-inositol-4,5-bisphosphate (IP2) are shown as blue and yellow dashed lines, respectively. Red and blue colors indicate negatively and positively charged amino acids. Note, lack of interaction with IP2 in the mutated (Arg621His) protein

The 3D visualization of wild-type (WT) and mutated (Arg621His) structures of the PLCB4 active site revealed the lack of interaction with IP2 in the mutated protein as compared to the native molecule (Fig. 3b) (Hicks et al. 2008). Besides, we have shown the conservation of the p.Arg621 residue among eleven species and through bioinformatics scores such as the PhastCons100way (dbNSFP v4.0) score in vertebrate–1, PhyloP100way (dbNSFP v4.0) score in vertebrate–10.003, and Genomic Evolutionary Rate Profiling (GERP) score 6.17.

## Discussion

In the current study, we are describing another individual presenting with ACS2, which is an ultra-rare disease. The 8-year-old boy is the 4^th^ reported patient carrying the missense mutation p.Arg621His in the *PLCB4* gene, in addition to case M001 described by Rieder et al., case 6 by Gordon et al., and the familial cases III: 2, II: 4, III: 1, and II: 1 by Nabil et al. (Table 1) (Rieder et al. 2012; Gordon et al. 2013a, b; Nabil et al. 2020). We obtained the molecular diagnosis applying the trio WES approach. Since the coverage of *PLCB4* was only 80x and results indicated a possibility of somatic mosaicism in the index, we subjected the patient’s and his parents’ DNA to deep targeted NGS of a custom craniofacial disorders–associated gene panel (Supplementary File 1) with a mean coverage of 2000x. The data we have obtained, however, pointed to the heterozygosity of the p.Arg621His missense variant, which was further confirmed through Sanger sequencing (Fig. 2).

Interestingly, the alteration, which we have described, was the first causative mutation linked to ACS2 (Rieder et al. 2012). The clinical features resulting from this variant have been shown in Table 1. All patients present with micrognathia, prominent cheeks, facial asymmetry, asymmetric mandible, and question mark ears. Those features seem to be cardinal for ACS. Notably, none of the affected individuals had either a cleft palate or a hearing impairment. Three individuals, however, displayed obstructive apnea, which has been described as an unusual feature of ACS2. Another interesting finding is the presence of microstomia and full mouth at the same time (Table 1; cases III: 2 and II: 4 vs. cases III: 1 and II: 1). The clinical spectrum of this Mendelian disease is heterogeneous and may overlap with other syndromes that result from the developmental defects of the first and second pharyngeal arches. Hence, the precise dysmorphological assessment in those patients constitutes a crucial stage of genetic counseling.

ACS2 inherits in an autosomal dominant manner and has been linked to thirteen heterozygous missense mutations in the *PLCB4* gene, so far. All those point variants occur within highly conserved catalytical X and Y domains of phospholipase C beta 4 protein (Fig. 3a) (Leoni et al. 2016; Rieder et al. 2012; Gordon et al. 2013a, b; Romanelli Tavares et al. 2017; Kido et al. 2013). However, three exceptional recessively inherited cases (two sporadic and one familial), presenting with a more severe phenotype and additional atypical symptoms, have been reported in the medical literature. First, Gordon et al. described an individual carrying a homozygous *PLCB4* alteration—c.1612-279_2015+1546del14997; p.Gly538Valfs*12—that was inherited from the healthy consanguineous heterozygous parents (Gordon et al. 2013a, b). Second, Kido et al. presented two siblings being compound heterozygotes for c.854-1G>A and c.1238+1G>C variants, inherited from the healthy non-consanguineous father and mother, respectively (Kido et al. 2013). Finally, the third patient, who was born to a healthy, consanguineous couple, harbored a homozygous deletion c.624delG; p.(Lys208Asnfs*5) (Leoni et al. 2016). Noteworthy, none of the stop gain, frameshift, splicing, and deletion type mutations was identified in heterozygosity among ACS2 cases. Screening of the gnomAD database, however, showed the presence of this type of alterations in a healthy cohort (Table 2). The above findings support the genetic concept of a negative dominance phenomenon according to which a mutated polypeptide with a missense alteration reduces the activity of co-expressed wild-type protein. Therefore, only the suppression of the two PLCB4 copies resulting either from truncating mutation on both alleles or dominant-negative effect exerted by a heterozygous missense variant on the wild-type allele would give rise to ACS2 phenotype. The main limitation of all *PLCB4* studies, however, seems to be the lack of functional analyses, which would allow for a better understanding of the data described above.

**Table 2:** An overview of stop gain and frameshift variants found in the *PLCB4* gene available in gnomAD browser database (accessed on 27 July 2020). Interestingly, one missense variant p.Arg621Cys associated with ACS2 was reported with the populational frequency of 0.000319

Variant ID	Consequence	Annotation	Allele frequency
20-9389726-C-T	p.Arg621Cys	Missense	3.19E−05
20-9404500-C-T	p.Arg797*	Stop gained	3.98E−06
20-9404515-C-T	p.Arg802*	Stop gained	3.98E−06
20-9404524-G-T	p.Gly805*	Stop gained	3.98E−06
20-9417677-CT-C	p.Ser870Profs*16	Frameshift	3.98E−06
20-9440284-CAA-C	p.Lys1014Argfs*20	Frameshift	4.01E−06
20-9449228-G-T	p.Glu1075*	Stop gained	3.98E−06
20-9449234-C-T	p.Arg1077*	Stop gained	3.98E−06
20-9453451-TAAAC-T	p.Asn1111Alafs*16	Frameshift	4.07E−06
20-9453986-G-T	p.Glu1145*	Stop gained	4.09E−06
20-9459570-G-T	p.Glu1167*	Stop gained	7.96E−06
20-9459583-C-T	p.Gln1159*	Stop gained	3.98E−06

Interestingly, we have found that one out of thirteen missense mutations—c.1861C>T p.Arg621Cys, which was described in an affected individual M003 and his unaffected father, also occurs in a healthy cohort with a frequency of 0.00031. The data come from the genome sequencing, whereas the variant was noted among the African population. According to our in-house WES data, the variant was not present in 100 Polish controls, similarly to 1200 control exomes, filtered by Rieder et al. (2012). The gnomAD data associated with the presence of the unaffected carrier, however, may underpin the hypothesis of p.Arg621Cys incomplete penetrance.

To conclude, with this report, we have increased the total number of all molecularly confirmed ACS cases to 30. Noteworthy, the majority of those patients carry pathogenic variants within the *PLCB4* gene that cause ACS2. As mentioned above, the syndrome presents high phenotypic variability among and between affected families that impedes reaching an exact and unequivocal clinical and molecular diagnosis. Thus, describing additional clinically and molecularly well-characterized cases with variable phenotypes, even if carrying previously reported mutations, is essential for the increase of our knowledge in such orphan Mendelian diseases.

## Electronic supplementary material

ESM 1 (XLSX 8 kb).

## Data Availability

The data that support the findings of this study are available from the corresponding author upon reasonable request.
